# A structured assessment of emergency and acute care providers in Afghanistan during the current conflict

**DOI:** 10.1186/s12245-015-0069-0

**Published:** 2015-07-04

**Authors:** Leeda Rashid, Edris Afzali, Ross Donaldson, Paul Lazar, Raghnild Bundesmann, Samra Rashid

**Affiliations:** Mclaren Regional Medical Center Department of Family Medicine, Michigan State University College of Human Medicine, 401 South Ballenger Highway, Flint, MI 48532 USA; Harbor-UCLA Medical Center, Division of Emergency Medicine, 1000 West Carson Street, Torrance, California 90509 USA; Department of Emergency Medicine, Michigan State University/Synergy Medical Education Alliance, 1000 Houghton Ave, Saginaw, MI 48602 USA

## Abstract

**Background:**

Afghanistan has struggled with several decades of well-documented conflict, increasing the importance of providing emergency services to its citizens. However, little is known about the country’s capacity to provide such care.

**Methods:**

Three native-speaking Afghan-American physicians performed an assessment of emergency care via combined quantitative and qualitative survey tools. Hospitals in Kabul, Afghanistan were selected based on probability proportional to size methodology, in which size was derived from prior work in the country and permission granted by the administering agency and the Ministry of Health. A written survey was given to physicians and nurses, followed by structured focus groups, and multiple days of observation per facility. A descriptive analysis was performed and data analyzed through a combination of variables in eight overarching categories relevant to emergency care.

**Results:**

One hundred twenty-five surveys were completed from 9 hospitals. One third of respondents (32.8 %) worked full time in the emergency departments, with another 28.8 % working there at least three quarters of the time. Over 63 % of providers believed that the greatest delay for care in emergencies was in the prehospital setting. Differences were noted among the various types of facilities when looking at specific components of emergency care such as skill level of workers, frequencies of assaults in the hospitals, and other domains of service provision. Sum of squares between the different facility types were highest for areas of skill (SS = 210.3; *p* = .001), confidence in the system (SS = 156.5; *p* < .005), assault (SS = 487.6; *p* < .005), and feeling safe in the emergency departments (SS = 193.1, *p* < .005). Confidence negatively correlated to frequency of assaults (Pearson *r* = −.33; *p* < .005) but positively correlated with feeling safe (Pearson *r* = .51; *p* < .005) and reliability of equipment (Pearson *r* = .48; *p* < .005). The only correlation for access to services was prehospital care (Pearson *r* = .72, *p* < .005).

**Conclusions:**

There is a significant need to provide emergency care services in Afghanistan, specifically prehospital care. High variability exists among facility-type in various components of emergency services provision.

**Electronic supplementary material:**

The online version of this article (doi:10.1186/s12245-015-0069-0) contains supplementary material, which is available to authorized users.

## Background

In early 2002, the Ministry of Public Health (MoPH) of Afghanistan and the major donor organizations for the country, including the World Bank, the United States Agency for International Development, and the European Commission, created a Basic Package of Health Services (BPHS) for Afghanistan [1]. The BPHS was a multilateral approach to help address the most pressing health issues in the country and was the first time a low-income nation implemented such a comprehensive package while in the midst of conflict [2]. The Essential Package of Hospital Services (EPHS) followed suit to standardize how hospitals were to be staffed, organized, and equipped for care and as referral centers for the BPHS [3].

The World Bank has advocated for designing essential packages of health services based on country-specific burden of disease for some time [4]. Afghanistan’s package aimed in part to alleviate the uncoordinated and often separate objectives of health care delivery by non-governmental organizations (NGOs), a problem exacerbated by the more than 30 years of conflict in the country [5]. Both the BPHS and EPHS created a universal set of health services to be delivered, focusing heavily on maternal and child mortality [6]. It additionally gave leadership of the BPHS/EPHS to the MoPH, while allowing for health service delivery to be contracted out to international NGOs that were already established and providing care [7]. Since its implementation, the BPHS/EPHS has generally thought to be effective in both capacity building and service delivery, with Afghanistan’s performance measure scorecard showing its citizens receiving more health services since its implementation in 2002 [8, 9].

However, despite improvements in general and preventive health outcomes [10, 11], tertiary and specialty hospitals still only receive 26 % of the total funds allocated to the MoPH from government [12]. This leaves most of the tertiary hospitals with poor facility infrastructure, an inadequate workforce, and lack of necessary supplies [13]. It is also noteworthy that Afghanistan’s health system is largely dependant on foreign aid and a large portion of health services provisions are contracted out to NGO’s [14].

Afghanistan’s general health structure since the implementation of the BPHS/EPHS contains little recommendations regarding the establishment of emergency and acute care for the country [15]. This is despite the analysis showing that acute illness and injuries rank among the highest causes of death and disability adjusted life-years (DALYs) lost in low- and middle-income countries [16]. Given the long history of conflict in Afghanistan, emergency systems of care are arguably even more important in this context. Given the lack of a formal emergency system, the paucity of research about acute care, and ongoing conflict in the country, little is known about the current provision of emergency care in Afghanistan. We therefore designed and implemented a survey to analyze knowledge, attitudes, and practice among clinicians providing emergency-related care in the country.

## Methods

### Data gathering

We chose a convenience sampling of hospital physicians, nurses and medical residents in training to survey in Kabul, Afghanistan. All hospitals were either public, private, or run by the Afghan military. Approval was granted from the McLaren IRB/Ethics Review Board for exemption status and the Ministry of Health of Afghanistan. For each chosen hospital, we provided a written survey to providers that were on duty on sequential days. Provider inclusion criteria were physicians, residents, or nurses trained in Afghanistan, employed by the institutions and willing to answer our written survey.

The written survey was an 87-item questionnaire in Likert-scale focusing on personnel background and training, hospital background, emergency room services, emergency personnel, transportation, and prehospital questions (Additional file 1). It combined elements from two previously validated tools: an emergency medicine assessment used in Iraq by Donaldson et al. [17] and the World Health Organization’s Tool for Situational Analysis to Assess Emergency and Surgical Care [18].

After completion of the written survey, we held focus group discussions with approximately ten providers at each of the hospitals. The oral questions (Additional file 2) were open-ended and emergency written.

To maintain a heterogeneity of opinions, we opted to interview groups of physicians, nurses, and resident physicians.

Finally, we spent around 7 days visiting each hospital to observe the triage and emergency care systems in practice. During each visit, we spoke with key informants, including administrative officials, chiefs of staff, and hospital executives. These observations were either recorded in audio or via written notes.

### Analysis

After collection, the data was coded and entered into SPSS software. Quality checks were performed on every tenth entry. We used PASW 18 Statistical Package (PASW Statistics 18, www.spss.com) for data analysis.

After completing initial descriptive analysis, we coded the written survey questions into eight overarching categories relevant to the practice of emergency medicine in the country (Additional file 3).

We then used these categories to compare differences in responses between government, NGO, and Public hospitals’ personnel using a two-way ANOVA. We did this because we could not control for the myriad of other factors such as location within the city, popularity of the facility, and ease of access to the facility.

To elucidate correlations between the summary measures, we used Kendall’s Tau-b method, since some of the data were not normally distributed. We additionally ran Pearson’s correlations on the same data and the results confirmed similar and significance levels.

## Results

There were 125 surveys returned: 62 (49.6 %) from government hospitals, 42 (33.6 %) from military hospitals, 17 (13.6 %) from NGO hospitals, and 4 (3.2%) not specified (Table 1). More than half of the respondents were physicians and another quarter were nurses. Of our 125 respondents, 34.7 % stated they worked in an emergency room-type area full time, 88.7 % said they had some form of life support training, and 55.4 % said they had ACLS training.

**Table 1:** Frequency table for baseline descriptives

	# (%) Respondents
Professional category	
No answer	1 (0.8)
Doctor	83 (66.4)
Nurse	28 (22.4)
Resident	13 (10.4)
Total	125
Do you currently work only in emergency section	
Yes	89 (71.2)
No	31 (24.8)
What percentage of your current clinical practice do you spend in the emergency section?	
No answer	1 (0.8)
1–10 %	10 (8)
11–25 %	14 (11.2)
26–50 %	16 (12.8)
51–75 %	28 (22.4)
76–99 %	8 (6.4)
100 %	41 (32.8)
What type of hospital	
Government non teaching	37 (29.6)
Private non teaching	12 (9.6)
Government teaching hospital	57 (45.6)
Private teaching hospital	14 (11.2)
Where do you see the greatest delay for care in emergencies?	
Prehospital	79 (63.2)
Waiting room	6 (4.8)
In the emergency section waiting for room	14 (11.2)
On the medicine/surgery floors	1 (0.8)
Do you feel emergency care should be included in the BPHS/EPHS	
No answer	2 (1.6)
Yes	57 (45.6)
No	53 (42.4)
Necessary equipment is immediately available for use during emergencies	
Strongly agree	71 (56.8)
Agree	43 (34.4)
Neutral	1 (0.8)
Strongly disagree	1 (0.8)
Improve nurse training	
No	61 (48.8)
Yes	52 (41.6)
What is average time to get to hospital in emergency	
<5 min	2 (1.6)
5–30 min	23 (18.4)
31 to 60 min	46 (36.8)
61–120 min	14 (11.2)
121–180 min	11 (8.8)
>3 h	14 (11.2)
Is there a universal phone number to call to get an ambulance in your area?	
Yes	104 (83.2)
No	9 (7.2)
No answer	2 (1.6)
If you called this phone number, how long on average does it take an ambulance to arrive?	
<5 min	2 (1.6)
5–30 min	51 (40.8)
31 to 60 min	44 (35.2)
61–120 min	11 (8.8)
>3 h	1 (0.8)
If a family member became seriously ill at home, how would you seek medical care?	
Keep comfortable treat at home	3 (2.4)
Wait for doc to arrive at home	1 (0.8)
Carry to hospital	43 (34.4)
Transport via private car or taxi	40 (32)
Call for ambulance	29 (23.2)
If a family member became seriously ill outside the home, how would you seek medical care?	
Keep comfortable treat at home	2 (1.6)
Wait for doc to arrive at home	4 (3.2)
Carry to hospital	66 (52.8)
Transport via private car or taxi	28 (22.4)
Call for ambulance	14 (11.2)
There is a need for emergency med as specialty	
Strongly agree	73 (58.4)
Agree	48 (38.4)
Neutral	3 (2.4)
Where do you see the greatest delay for care in emergencies?	
No answer	1 (0.8)
Prehospital	79 (63.2)
Waiting room	6 (4.8)
In the emergency section waiting for room	14 (11.2)
Total	100 (80)

Table 1 reveals the general makeup of the health workers in our survey and their attitudes toward various components of emergency care. 76.8 % of our respondents were either physicians or physicians in training. 61.6 % of respondents worked half to full time in the ED. The majority worked at government teaching and non teaching hospitals. Overall, our respondents agreed that the greatest obstacle/delay to getting health in an emergency situation was prehospital care (63.2 %). The majority of respondents noted it would take between 30 and 60 min to wait for the arrival of an ambulance and to get to the hospital. Close to two-thirds of our respondents noted that if family members were to get ill, it was best to bring them via private car or even carry them, instead of calling an ambulance. Eighty-three percent admitted that there was a reliable number to call for help; however, despite this, respondents consistently noted that they would rather take their loved ones by private car or taxi. Over 96 % surveyed agreed that emergency medicine needs to be prioritized as a specialty.

We then coded remaining questions into the following overarching emergency care relevant, aggregated variables:

1. Emergency procedural skills
2. Confidence in hospital emergency care
3. ED safety
4. Assault on personnel in the ED
5. Staffing issues
6. Equipment and supplies
7. Access to emergency care
8. Prehospital care and transport time

A comparison of the means for the aggregated categories (Table 2) showed that the highest skill level was in the NGO hospitals; military hospitals had the second best skill level and the MoPH had the lowest skill level. For adequacy of staffing, the NGOs again were the best staffed, the military was second, and the MoPH again had the lowest staffing.

**Table 2:** Comparison of means between hospital types

Descriptive statistics						
What type of hospital		*N*	Minimum	Maximum	Mean	Std. deviation
		Statistic	Statistic	Statistic	Statistic	Statistic
Military	Assault	39	4	16	7.97	3.483
	Confidence	40	9	15	12.7	1.488
	Access	33	6	29	9.48	4.258
	Equipment	40	6	10	8.03	1.165
	Feel safe	39	12	20	15.31	1.962
	Prehospital	35	4	11	6.23	2.03
	Skill	34	9	20	15.24	3.542
	Staff	39	4	10	6.64	1.98
	Valid N (listwise)	27				
NGO	Assault	17	4	15	5.71	3.46
	Confidence	16	10	15	13	1.265
	Access	14	6	13	9.14	1.916
	Equipment	17	6	10	7.82	1.131
	Feel Safe	16	13	20	16.75	2.145
	Prehospital	14	4	9	6.14	1.406
	Skill	16	9	20	16.31	3.049
	Staff	17	5	10	7.65	1.057
	Valid *N* (listwise)	12				
MOPH	Assault	49	4	22	11.33	4.819
	Confidence	53	6	15	10.4	2.133
	Access	45	5	13	8.84	1.918
	Equipment	52	2	8	6.5	1.502
	Feel Safe	51	4	17	13.2	2.417
	Prehospital	49	3	9	6.04	1.485
	Skill	47	6	20	12.72	4.025
	Staff	55	2	8	5.51	1.597
	Valid N (listwise)	35				

The military hospital felt their equipment was most adequate, followed by NGOs, then the MoPH Hospitals. In the feeling safe component, the NGOs felt they were the safest, while the military ranked second and the MoPH hospitals rated lowest. Assaults were also most common in the MoPH hospitals, least in the NGO’s and the military again ranked in the middle.

When we used two-way ANOVA on these summary measures, to understand if the differences between the facilities for each measure were significant, we found statistically significant differences among the facilities with regard to each summary measure we had defined as being core components of emergency care; concluding that differences of opinion were not likely random. The only summary measures that were not statistically significantly different among the various facilities were access and prehospital care (Table 3). Since some of our data was dichotomous, some might argue that we did not meet assumptions, but we felt differently because we have the means of mixed data. However, to confirm these findings, we also ran the nonparametric equivalent, Kruskal-Wallis analysis, and the results largely coincided (Table 4).

**Table 3:** Analysis of variance among summary measures

ANOVA						
		Sum of squares	df	Mean square	*F*	Sig.
Staff	Between groups	69.308	2	34.654	12.287	0
	Within groups	304.602	108	2.82		
	Total	373.91	110			
Access	Between groups	7.817	2	3.908	0.44	0.645
	Within groups	789.868	89	8.875		
	Total	797.685	91			
Prehospital	Between groups	0.727	2	0.363	0.127	0.881
	Within groups	271.804	95	2.861		
	Total	272.531	97			
Equipment	Between groups	58.839	2	29.419	16.548	0
	Within groups	188.446	106	1.778		
	Total	247.284	108			
Feel safe	Between groups	193.144	2	96.572	19.606	0
	Within groups	507.347	103	4.926		
	Total	700.491	105			
Assault	Between groups	487.635	2	243.818	14.072	0
	Within groups	1767.279	102	17.326		
	Total	2254.914	104			
Confidence	Between groups	156.499	2	78.249	23.898	0
	Within groups	347.079	106	3.274		
	Total	503.578	108			
Skill	Between groups	210.319	2	105.159	7.61	0.001
	Within groups	1298.959	94	13.819		
	Total	1509.278	96

**Table 4:** Post hoc tests (nonparametric Kruskal-Wallis)

	Multiple comparisons			
Tukey HSD				
Dependent variable	(I) What type of hospital	(J) What type of hospital	Mean difference (I-J)	Sig.
Staff	Military	NGO	−1.006	0.103
		MOPH	1.132^*^	0.005
	NGO	Military	1.006	0.103
		MOPH	2.138^*^	0
	MOPH	Military	−1.132^*^	0.005
		NGO	−2.138^*^	0
Access	Military	NGO	0.342	0.931
		MOPH	0.64	0.618
	NGO	Military	−0.342	0.931
		MOPH	0.298	0.943
	MOPH	Military	−0.64	0.618
		NGO	−0.298	0.943
Prehospital	Military	NGO	0.086	0.986
		MOPH	0.188	0.871
	NGO	Military	−0.086	0.986
		MOPH	0.102	0.978
	MOPH	Military	−0.188	0.871
		NGO	−0.102	0.978
Equipment	Military	NGO	0.201	0.861
		MOPH	1.525^*^	0
	NGO	Military	−0.201	0.861
		MOPH	1.324^*^	0.002
	MOPH	Military	−1.525^*^	0
		NGO	−1.324^*^	0.002
Feel safe	Military	NGO	−1.442	0.078
		MOPH	2.112^*^	0
	NGO	Military	1.442	0.078
		MOPH	3.554^*^	0
	MOPH	Military	−2.112^*^	0
		NGO	−3.554^*^	0
Assault	Military	NGO	2.268	0.151
		MOPH	−3.352^*^	0.001
	NGO	Military	−2.268	0.151
		MOPH	−5.621^*^	0
	MOPH	Military	3.352^*^	0.001
		NGO	5.621^*^	0
Confidence	Military	NGO	−0.3	0.841
		MOPH	2.304^*^	0
	NGO	Military	0.3	0.841
		MOPH	2.604^*^	0
	MOPH	Military	−2.304^*^	0
		NGO	−2.604^*^	0
Skill	Military	NGO	−1.077	0.606
		MOPH	2.512^*^	0.01
	NGO	Military	1.077	0.606
		MOPH	3.589^*^	0.003
	MOPH	Military	−2.512^*^	0.01
		NGO	−3.589^*^	0.003

*The mean difference is significant at the 0.05 level

To understand if there was any correlations among our summary measures, we ran both Kendall Tau B correlations and Pearson’s correlations between the summary measures (two-tailed) and some of our measures were significant at the .01 to .05 level.

Skill level was significantly correlated to the type of hospital, confidence in the benefit of emergency care, feeling safe while practicing, and having sufficient supplies (Tables 5 and 6).

**Table 5:** Correlations (Pearson’s)

Correlations										
		Confident	Assault	Feel safe	Equipment	Prehospital	Access	Staffing	Skill	What type of hospital
Confidence	Pearson Correlation	1	–0.336**	0.515**	0.481**	−0.1	−0.058	0.322**	0.469**	0.260**
	Sig. (two-tailed)		0	0	0	0.326	0.581	0.001	0	0.007
	Sum of squares and cross-products	504.124	−320.365	277.077	156.783	−33.694	−31.29	136.622	362.787	62.972
	Covariance	4.501	−3.11	2.69	1.493	−0.347	−0.34	1.242	3.901	0.589
	*N*	113	104	104	106	98	93	111	94	108
Assault	Pearson correlation	−0.336**	1	−0.547**	−0.438**	0.087	−0.02	−0.312**	−0.069	−0.221*
	Sig. (two-tailed)	0		0	0	0.398	0.851	0.001	0.52	0.024
	Sum of squares and cross-products	−320.365	2308.807	−676.874	−315.514	64.608	−24.446	−284.557	−99.622	−109.077
	Covariance	−3.11	21.378	−6.636	−3.034	0.673	−0.269	−2.71	−1.119	−1.059
	*N*	104	109	103	105	97	92	106	90	104
Feel safe	Pearson correlation	0.515**	−0.547**	1	0.716**	−0.271**	−0.205	0.415**	0.258*	0.207*
	Sig. (two-tailed)	0	0		0	0.007	0.05	0	0.014	0.035
	Sum of squares and cross-products	277.077	−676.874	706.972	299.157	−114.371	−145.304	210.896	220.798	56.952
	Covariance	2.69	−6.636	6.546	2.796	−1.191	−1.597	2.009	2.509	0.553
	*N*	104	103	109	108	97	92	106	89	104
Equipment	Pearson correlation	0.481**	−0.438**	0.716**	1	−0.18	−0.125	0.324**	0.220*	0.111
	Sig. (two-tailed)	0	0	0		0.076	0.231	0.001	0.035	0.256
	Sum of squares and cross-products	156.783	−315.514	299.157	248.857	−43.837	−51.723	96.843	111.011	18.093
	Covariance	1.493	−3.034	2.796	2.242	−0.452	−0.556	0.905	1.22	0.171
	*N*	106	105	108	112	98	94	108	92	107
Prehospital	Pearson correlation	−0.1	0.087	−0.271**	−0.18	1	0.719**	−0.211*	−0.097	0.007
	Sig. (two-tailed)	0.326	0.398	0.007	0.076		0	0.036	0.372	0.947
	Sum of squares and cross-products	−33.694	64.608	−114.371	−43.837	298.912	336.747	−68.061	−55.605	1.155
	Covariance	−0.347	0.673	−1.191	−0.452	2.96	3.582	−0.694	−0.654	0.012
	*N*	98	97	97	98	102	95	99	86	97
Access	Pearson correlation	−0.058	−0.02	−0.205	−0.125	0.719^**^	1	−0.104	−0.163	0.119
	Sig. (two-tailed)	0.581	0.851	0.05	0.231	0		0.323	0.139	0.266
	Sum of squares and cross-products	−31.29	−24.446	−145.304	−51.723	336.747	820.905	−54.462	−112.095	32.222
	Covariance	−0.34	−0.269	−1.597	−0.556	3.582	8.733	−0.592	−1.351	0.362
	*N*	93	92	92	94	95	95	93	84	90
Staffing	Pearson correlation	0.322**	−0.312**	0.415**	0.324**	−0.211*	−0.104	1	0.106	0.272**
	Sig. (two-tailed)	0.001	0.001	0	0.001	0.036	0.323		0.313	0.004
	Sum of squares and cross-products	136.622	−284.557	210.896	96.843	−68.061	−54.462	393.183	73	57.273
	Covariance	1.242	−2.71	2.009	0.905	−0.694	−0.592	3.449	0.793	0.525
	*N*	111	106	106	108	99	93	115	93	110
Skill	Pearson correlation	0.469**	−0.069	0.258*	0.220*	−0.097	−0.163	0.106	1	0.068
	Sig. (two-tailed)	0	0.52	0.014	0.035	0.372	0.139	0.313		0.51
	Sum of squares and cross-products	362.787	−99.622	220.798	111.011	−55.605	−112.095	73	1510.634	26.667
	Covariance	3.901	−1.119	2.509	1.22	−0.654	−1.351	0.793	15.106	0.281
	*N*	94	90	89	92	86	84	93	101	96
What type of hospital	Pearson correlation	0.260**	−0.221*	0.207*	0.111	0.007	0.119	.272^**^	0.068	1
	Sig. (two-tailed)	0.007	0.024	0.035	0.256	0.947	0.266	0.004	0.51	
	Sum of squares and cross-products	62.972	−109.077	56.952	18.093	1.155	32.222	57.273	26.667	130.8
	Covariance	0.589	−1.059	0.553	0.171	0.012	0.362	0.525	0.281	1.099
	*N*	108	104	104	107	97	90	110	96	120

*Correlation is significant at the 0.05 level (2-tailed); **Correlation is significant at the 0.01 level (two-tailed)

**Table 6:** Nonparametric correlations (Kendall Tau)

Correlations											
			Confidence	Assault	Feel Safe	Equipment	Prehospital	Access	Staff	Skill	What type of hospital
Kendall’s tau_	Confidence	Correlation coefficient	1	−0.248**	0.390**	0.416**	−0.085	−0.086	0.253**	0.387**	0.170*
		Sig. (two-tailed)		0.001	0	0	0.28	0.283	0.001	0	0.032
		*N*	113	104	104	106	98	93	111	94	108
	Assault	Correlation coefficient	−0.248**	1	−0.471**	−0.313**	0.056	0.023	−0.249**	−0.046	−0.192*
		Sig. (two-tailed)	0.001		0	0	0.468	0.763	0.001	0.549	0.014
		*N*	104	109	103	105	97	92	106	90	104
	Feel safe	Correlation coefficient	0.390**	−0.471**	1	0.547**	−0.189*	−0.225**	0.311**	0.151	0.202*
		Sig. (two-tailed)	0	0		0	0.017	0.005	0	0.059	0.012
		*N*	104	103	109	108	97	92	106	89	104
	Equipment	Correlation coefficient	0.416**	−0.313^**^	0.547**	1	−0.171*	−0.183*	0.224**	0.183*	0.097
		Sig. (two-tailed)	0	0	0		0.041	0.029	0.006	0.027	0.247
		*N*	106	105	108	112	98	94	108	92	107
	Prehospital	Correlation coefficient	−0.085	0.056	−0.189*	−0.171*	1	0.835**	−0.145	−0.072	0.008
		Sig. (two-tailed)	0.28	0.468	0.017	0.041		0	0.071	0.374	0.923
		*N*	98	97	97	98	102	95	99	86	97
	Access	Correlation coefficient	−0.086	0.023	−0.225^**^	−0.183^*^	0.835**	1	−0.156	−0.111	0.028
		Sig. (two-tailed)	0.283	0.763	0.005	0.029	0		0.055	0.17	0.743
		*N*	93	92	92	94	95	95	93	84	90
	Staffing	Correlation coefficient	0.253**	−0.249**	0.311**	0.224**	−0.145	−0.156	1	0.082	0.244**
		Sig. (two-tailed)	0.001	0.001	0	0.006	0.071	0.055		0.301	0.002
		*N*	111	106	106	108	99	93	115	93	110
	Skill	Correlation coefficient	0.387**	−0.046	0.151	0.183*	−0.072	−0.111	0.082	1	0.049
		Sig. (two-tailed)	0	0.549	0.059	0.027	0.374	0.17	0.301		0.547
		*N*	94	90	89	92	86	84	93	101	96
	What type of hospital	Correlation coefficient	0.170*	−0.192*	0.202*	0.097	0.008	0.028	0.244**	0.049	1
		Sig. (two-tailed)	0.032	0.014	0.012	0.247	0.923	0.743	0.002	0.547	
		*N*	108	104	104	107	97	90	110	96	120

*Correlation is significant at the 0.05 level (2-tailed); **Correlation is significant at the 0.01 level (two-tailed)

Confidence in the particular health system was negatively correlated to frequency of assaults, and positively correlated with feeling safe in that particular system and in the adequacy of supplies/equipment, the amount of hospital staffing and in the skill level of the workers (Table 6).

Time to the ED/prehospital time was heavily correlated to the levels of access in each facility.

The type of hospital was significantly correlated to the number of assaults experienced by respondents and negatively correlated to confidence in the system, feeling safe, adequacy of staffing, and supplies.

From our observations, medical training and adequate equipment was a large barrier in providing services. Many public facilities were often so crowded that we could not safely get in through the hospital doors and we met many patients waiting hours for basic life support mechanisms such as oxygen tanks or an EKG. Physicians were very eager to learn, and requested support for medical education and greater training. User fees were collected at one of the NGO sites, whereas all other facilities collected intermittently for medical supplies, blood products, and other equipment that were not immediately available within the facility itself.

## Discussion

The Afghan health care system is limited in its capacity to provide in-and-out-of-hospital emergency care. Our data and analysis shows wide variation in emergency services provided in Kabul, with much of the variability dependent on the type of hospital facility.

We found that medical training at the military hospital had some, although limited focus on emergency medical training, but this was not persistent in the public sector system. Resident physicians often noted that they were left to deal with emergencies that came to the hospital, regardless of whether they had prior training in certain clinical scenarios. During focus groups and in our observations, limitations highlighted were not always due to resources constraints but also to a lack of organizational structure and processes in place to prioritize and triage cases.

Although the public hospitals see a disproportionately large number of patients, they trended toward having less capacity, supply, and resources. These issues were highlighted in their level of confidence, skill and staffing issues corroborated by our quantitative analyses. They were also more subject to frequent assaults and disaster scenarios, further creating barriers to consistent staffing of the emergency departments. From our own observations, their medical and equipment supply, including items as simple as oxygen supplementation, did not meet the demands of the volume of patients treated daily.

From our observations, the military hospitals were not open for civilian care unless injuries were the direct result of combat. Our focus groups highlighted that most patients, despite being from remote areas of the provinces, knew of the existence of public facilities and were either referred to or directly came to public facilities much more readily than the NGO hospitals or other private facilities. There was also a sense that public sector hospitals were always free, whereas NGO facilities would charge a fee, even though only one of the NGO facilities we visited had begun a process of user fee collection on a very limited basis.

From our summary measures and correlations data, we found that NGO’s consistently had the better trained staff compared to the public and military hospital. We found that confidence in emergency medicine skills, such as intubation were much better in NGO and military hospitals as compared to the Public system. Even when we split the data based on occupation (nurse or physician), we found that differences among the facilities persisted in their level of skill.

Skill was also an outstanding variable that was positively correlated to the type of hospital (public, NGO, or military), confidence in the emergency care system, feeling safe while practicing, and having sufficient supplies. It is a possibility that the skilled workforce migrates to higher paying, better supplied, and safer working conditions.

Our assessment demonstrated that there is a high need for ongoing investment in the skills based training of physicians, nurses, residents, and other emergency personnel especially in the public sector hospitals.

Tables 5 and 6 show that the type of hospital was significantly correlated to the number of assaults experienced by respondents and negatively correlated to confidence in the system, feeling safe, and adequacy of staffing and supplies.

As part of the current debate on a national salary policy, adequate compensation, and incentives for health workers should be addressed to maintain adequate staffing for the care of emergency patients in light of such safety issues. Its also noteworthy that the reality of Kabul still purports a more secure work environment in comparison to other provinces and in particular rural areas where corruption will more likely be a contributing factor given fewer civil services and the paucity of security forces.

Time to the ED and prehospital time was heavily correlated to the levels of access in each facility. Whether this implied that more efficient prehospital care, as provided for example in places like the military, also allows for quicker and more effective initial entry and triage via the ambulance system cannot be determined by our quantitative data alone, but this was corroborated repeatedly by our focus group discussions and our qualitative analyses. Our own observations of seeing patients brought in by local taxis to the public hospitals also begged the question of whether more focus on the development of the prehospital system is a key to increasing access for all citizens. Time to ED undoubtedly differs in urban centers like Kabul, versus rural Afghanistan, but we cannot make any specific conclusions at this time.

Since most admitted knowing colleagues who were assaulted or having been assaulted themselves during the highly emotional moments that medical emergencies provoke, the addition of further security measures for workers in the hospitals, especially within the public sector hospitals, would allow physicians to feel safer committing more time to emergency sections, such as taking night shifts. Studies in Iraq have found that within the Emergency Department alone, over 80 % of physicians were victims of assault at least once [19]. Correlations found regarding safety do not again prove causality, but does confirm that workers within conflict zones are being threatened regularly, but in fact may be more willing to commit to night shifts and other less than ideal working conditions if they at least feel safe while there.

It is also worthwhile to discuss our two summary measures that were consistently not significantly different among facility type; access and prehospital care. Neither of these components proved to be different among the various facility types throughout our analyses. This may be indicative of the fact that most respondents were in agreement regarding the landscape of prehospital care and access issues. Therefore, when we tried to decipher if respondents felt differently about these particular issues based on their facility type, our conclusions were never statistically different.

## Conclusions

The challenges of providing care in Afghanistan combine those of a developing nation, an intra-conflict nation and a combat zone [20]. Our conclusions are that Afghanistan’s system of emergency and acute care is exposed to all of these challenges. Given the significant reliance on foreign aid, resource utilization, the limitations of unsustainable contracting mechanisms [21], and evidence based priority setting in service provision is paramount to delivering care.

Our survey combined with the focus group conclusions and our own first hand witness of the emergency system in Kabul, Afghanistan reveals critical lack of resources, capacity, and safety while providing initial care. Additionally, there is a widely accepted opinion that although an emergency call number exists, there is no consistent and reliable predhospital system.

There are frequent shortages of lifesaving medications, a lack of functioning medical equipment, and a paucity of opportunity for continuing training and medical education.

There are also few incentives for clinicians to provide emergency care. Neither the national health service primary package, the BPHS, or the hospital wide quality initiatives of the EPHS focus much detail on initial point of care guidelines or resources [12, 22]. At the hospital level, there appears to be little organizational structure for the triage of emergency patients. This places a high demand on physicians in other specialties who do not feel confident in the system, especially in the public sector. Clinicians providing emergency care are not comfortable if they have not had formal training in emergency services and are not confident in their skills toward some procedures, and they have to do their job in an environment with poorly functioning equipment and scarce medications. Combined with disincentives such as violence at work and poor pay by the public system, it is understandable that many providers choose not to make emergency care a priority.

In a country plagued by decades of war and unrest, a comprehensive and effective prehospital and emergency care system is paramount to saving lives, meeting critical health care needs, and providing a reliable safety net for the population.

Despite documented success in indicators as maternal mortality rates and infant mortality rates [23], much more needs to be done in meeting the needs of basic emergency services in a country that sees such acute events almost daily. Our data supports the need for focused efforts to improve prehospital and hospital-based care in Afghanistan, starting with the inclusion of emergency services training and organizational structures as a part of the expansion of basic health services in the country.

### Limitations

The major limitation in this study is that although the hospitals were randomly sampled, we used a convenience sampling of health workers present in the hospitals during our data collection period. The experience and opinions of the health workers present may not reflect those of their hospital overall throughout the full year.

Additionally, our study was limited to Kabul. Although a majority of resources are concentrated in Kabul, it is unclear if this survey is generalizable to other urban centers and the more remote areas of Afghanistan. As of 2012, there are still only 26 hospitals in the entire country that implement the quality initiatives and standards of the EPHS, therefore data on the capacity of emergency care in all hospitals as a whole may not be fully reflected at our chosen sites [12]. Kabul also has only one of its major hospitals implementing the EPHS that is funded and managed by the MoPH. The current contracts that support emergency services through the MoPH are also not in Kabul province, and therefore our results may not be generalizable to MoPH facilities providing acute care.

It is possible that our survey was influenced by cultural bias, since many health workers may fear job loss in an ongoing insecure labor market, due to retribution if respondents were honest about the shortcomings of the system. We maintained that all surveys were completely confidential, but this limitation is still a possibility. Also, though we did not ask directly about corruption and theft as a factor during open-ended focus groups, it is likely that out of fear of retribution or cultural nuances, these issues were not discussed.

In our focus groups, we avoided asking direct response questions and instead opted for more open-ended questions. However, it is a well-known limitation of focus groups that surveyors asking questions may indirectly elucidate responses already programmed through a society’s own belief system and systems of hierarchy (Maxwell) [24]. Focus groups were held in the most culturally appropriate way so as to elicit a sense of mutual respect and understanding for the goals of the project, thereby obtaining more critical thinking and obtaining increasing accuracy of information. However, this limitation must be considered regardless.

## Additional files

Additional file 1:
**Survey.**
Additional file 2:
**Focus Group Questionnaire.**
Additional file 3:
**Summary Measures Defined.**
