# Supplementary material for: A structured assessment of emergency and acute care providers in Afghanistan during the current conflict
Source: Int J Emerg Med. 2015 Jul 4;8:21. doi: 10.1186/s12245-015-0069-0 (PMC4495094; doi:10.1186/s12245-015-0069-0)
Supplement: Additional file 1: — Survey. [file 12245_2015_69_MOESM1_ESM.pdf]

Additional file 1

Survey

Training Background Questions

Please answer the following questions to the best of your abilities. Please try to give an answer for every question.

1. What is your job title?

2. Do you currently work in an emergency section?
   1. Yes
   2. No

3. Do you have clinical responsibilities outside of the emergency section (e.g. operating theater, outside specialty clinics, etc.)?
   1. Yes
   2. No

4. What percentage of your current clinical practice do you spend in the emergency section?
   1. 0%
   2. 1-10%
   3. 10-25%
   4. 25-50%
   5. 50-75%
   6. 75-99%
   7. 100%

5. How many years of training have you had after medical school/nursing school?
   1. None
   2. 1 year
   3. 2 years
   4. 3-4 years
   5. 5 or more years

6. Do you know what Basic Life Support, Advanced Cardiac Life Support, Advanced Trauma Life Support is?

1. Yes
2. No

7. Have you ever taken Basic Life Support or similar training?
    1. Yes
    2. No
8. Have you ever taken Advanced Cardiac Life Support (ACLS) or similar training?
    1. Yes
    2. No
9. Have you ever taken Advanced Trauma Life Support (ATLS) or similar training?
    1. Yes
    2. No
10. Do you currently hold a diploma or other special degree in emergency medicine?
    1. Yes
    2. No
11. Is your background training in:
    1. Emergency medicine (specialty training)
    2. General surgery
    3. Anesthesia
    4. Medicine
    5. Surgical sub-specialty
    6. Medical sub-specialty
    7. General practice
    8. No specialty training
12. Are you currently studying/training to become a physician/nurse in the emergency department?
    1. Yes
    2. No
13. Do you plan on taking any kind of board exams in emergency medicine?
    1. Yes
    2. No

14. Have you ever performed endotracheal intubation?
    1. Yes
    2. No
15. You currently feel comfortable performing endotracheal intubation if clinically indicated in an emergency.
    1. Strongly agree
    2. Agree
    3. Neutral
    4. Disagree
    5. Strongly disagree
16. You currently feel comfortable performing the initial resuscitation of a medical patient.
    1. Strongly agree
    2. Agree
    3. Neutral
    4. Disagree
    5. Strongly disagree
17. You currently feel comfortable performing the initial resuscitation of a trauma patient.
    1. Strongly agree
    2. Agree
    3. Neutral
    4. Disagree
    5. Strongly disagree
18. When a family member gets sick, where do you prefer to take them?
    1. Hospital
    2. Community clinic
    3. Private Clinic
    4. Home care
19. Patients will benefit from initial treatment in the emergency department prior to being transported to the medical/surgical floors.
    1. Strongly Agree
    2. Agree

3. Neutral
4. Disagree
5. Strongly Disagree

20. There is a need for emergency medicine as a specialty in Afghanistan.
    1. Strongly agree
    2. Agree
    3. Neutral
    4. Disagree
    5. Strongly disagree

21. Are you interested in the formation of an emergency medicine specialty society in Afghanistan?
    1. Very interested
    2. Interested
    3. Not sure
    4. Not interested
    5. Definitely not interested

Hospital Background Questions

22. What hospital do you primarily work at?

____

23. In what province is your hospital located?

____

24. What is the type of hospital?
    1. Government non-teaching hospital
    2. Private non-teaching hospital
    3. Government teaching hospital
    4. Private teaching hospital

25. What is the setting for this hospital?
    1. Rural
    2. Around a city (suburban)
    3. In a city (urban)

26. Estimate the percentage of your total income from:
   1. Government salary ________%
   2. Private patients ________%

27. Does your hospital specialize in a certain area of care (circle all that apply)
   1. Primary care
   2. Trauma/injury
   3. Surgery
   4. OB/GYN
   5. Pediatrics
   6. Infectious disease
   7. Burns
   8. Other (describe): ____________

28. Estimate mow many inpatient beds are available in this hospital?
   1. 0 - 20
   2. 21 – 50
   3. 51 – 100
   4. 101 – 250
   5. 251 – 1000
   6. >1000

29. Estimate how many people live in your town/area?
   1. 0 - 1,000
   2. 1,000 – 10,000
   3. 10,000 – 50,000
   4. 50,000 – 250,000
   5. 250,000 – 1,000,000
   6. >1,000,000

30. How many hospitals with emergency services are located in this town/area? _____

31. Where do you see the greatest delay to provide care for patients with emergencies?
   1. Pre-hospital
   2. Waiting room

3. In the emergency section (waiting for a room)
4. On the Medicine/Surgery floors

32. What do you think are the top

Tuberculosis	Cancer	Road Traffic Accidents
Diarrheal Disease	Diabetes mellitus	Poisonings
Pediatric Infections	Panic Disorder	Falls
Meningitis	Dementia	Fires
Hepatitis	Other Psychiatric (post traumatic stress disorder)	Drowning
Respiratory Infections	Headaches	Self-Inflicted Injuries
Maternal Conditions (pregnancy, intra-partum, post-partum)	Seizures	Bullet Injuries
Skin Disease	Cardiovascular Disease	Blast Injuries
Nutritional deficiencies	CVA	Back pain
Perinatal conditions (including neonatal death and infections)	Gastrointestinal Disease (including appendicitis, ulcers)	Genitourinary Diseases (including kidney stones, renal failure, urinary tract infections, pyelonephritis
HIV/AIDS	Arthritis	COPD/Asthma
STDs excluding HIV	Home Accidents (cooking, gas inhalation, etc.)	Inability to get to hospital (no ambulance; no escort; etc.)

33. What do you think are the top three reasons for *hospitalization* where you work? Please **circle three** from the options below:

Tuberculosis	Cancer	Road Traffic Accidents
Diarrheal Disease	Diabetes mellitus	Poisonings
Pediatric Infections	Panic Disorder	Falls
Meningitis	Dementia	Fires
Hepatitis	Other Psychiatric (post traumatic stress disorder)	Drowning

Respiratory Infections	Headaches	Self-Inflicted Injuries
Maternal Conditions (pregnancy, intra-partum, post-partum)	Seizures	Bullet Injuries
Skin Disease	Cardiovascular Disease	Blast Injuries
Nutritional deficiencies	CVA	Back pain
Perinatal conditions (including neonatal death and infections)	Gastrointestinal Disease (including appendicitis, ulcers)	Genitourinary Diseases (including kidney stones, renal failure, urinary tract infections, pyelonephritis
HIV/AIDS	Arthritis	COPD/Asthma
STDs excluding HIV	Home Accidents (cooking, gas inhalation, etc.)	Inability to get to hospital (no ambulance; no escort; etc.)

34. What do you think are the top three reasons for *visits to the emergency department* where you work? Please **circle three** from the options below:

Tuberculosis	Cancer	Road Traffic Accidents
Diarrheal Disease	Diabetes mellitus	Poisonings
Pediatric Infections	Panic Disorder	Falls
Meningitis	Dementia	Fires
Hepatitis	Other Psychiatric (post traumatic stress disorder)	Drowning
Respiratory Infections	Headaches	Self-Inflicted Injuries
Maternal Conditions (pregnancy, intra-partum, post-partum)	Seizures	Bullet Injuries
Skin Disease	Cardiovascular Disease	Blast Injuries
Nutritional deficiencies	CVA	Back pain
Perinatal conditions (including neonatal death and infections)	Gastrointestinal Disease (including appendicitis, ulcers)	Genitourinary Diseases (including kidney stones, renal failure, urinary tract infections, pyelonephritis

HIV/AIDS	Arthritis	COPD/Asthma
STDs excluding HIV	Home Accidents (cooking, gas inhalation, etc.)	Inability to get to hospital (no ambulance; no escort; etc.)

Emergency Section/Room Questions

35. How many emergency beds are available in this hospital? _______
36. What percentage of patients seen in the emergency department are younger than 18 years of age? ______%
37. What percentage of patients seen in the emergency department are greater than 65 years of age? ______%
38. What percentage of patients seen in the emergency department are then admitted to the hospital? _____%
39. Which specialty decides where a patient from the emergency department gets transferred to? __________________
40. Where do the majority of emergency patients come from?
    1. Home or in the community
    2. Doctor's office
    3. Other hospital
    4. Other (describe): _____________
41. Have you ever trained in a simulated environment?
    a. yes
    b. no
42. How long do patients wait on average to see a doctor after they get to the emergency department?

a. <5 minutes
b. 5 – 30 minutes
c. 31 – 60 minutes
d. 61 – 120 minutest
e. 121 – 180 minutes (2-3 hours)
f. >3 hours

43. Does your hospital currently have a trauma resuscitation team that responds to critical trauma patients?

a. Yes
b. No
c. Not sure

44. The emergency department at your hospital can be best described as:

a. Divided into medical and surgery sections only
b. Divided into medical, surgery, OB/GYN, and/or other sub-sections

c. Unified into one emergency department (with the exception of pediatrics)
d. Unified into one emergency department (including pediatrics)
e. There is no designated emergency department.

45. Is there a triage system to sort patients when they arrive to the emergency department?

a. Yes (we see patients based on severity of illness)
b. No (we see patients in order that they arrive to the hospital)
c. Not sure

46. If there is a triage system, who conducts the triage process (leave blank if you answered “No” to question #30)?

a. Nurse
b. Physician
c. Other (specify): ______________________

47. Do you know what Afghanistan’s Basic Package of Health Services (BPHS) is?

a. Yes
b. No

48.Do you feel the BPHS adequately addresses healthcare needs in your community?

a. Yes
b. No

49.Do you feel Emergency Care should be included in the BPHS?

a. Yes
b. No

50. How many disasters scenarios (bomb attack, earthquake, etc.) has your hospital faced in the last year?

a. None
b. 1-2
c. 2-4
d. 4-6
e. >6

51. Healthcare in Afghanistan has improved significantly since the BPHS was implemented.

a. Strongly Agree
b. Agree
c. Neutral
d. Disagree
e. Strongly Disagree

52. Our hospital is well prepared to cope with disasters (Bomb attack, earthquake, disease outbreak).

a. Strongly Agree
b. Agree
c. Neutral
d. Disagree
e. Strongly Disagree

53. Where do you feel the greatest improvements in disaster preparedness can come from?

a. Equipment supply
b. Nurse training
c. Physician training
d. Triage and management of a large influx of patients
e. No improvements needed

Emergency Personnel Questions

54. Who staffs the emergency department?

a. General practitioners
b. Resident doctors
c. Emergency medicine specialists
d. Other specialty physicians
e. Other (specify):___________________

55. How many years of medical training after medical school does a physician in your hospital need in order to work independently (at least for some period of time, for example at night) in the emergency department?

a. None
b. 1 year
c. 2 years
d. 3-4 years
e. 5 or more years

56. How many patients do physicians normally see in an average 8-hour period? _______

57. How many physicians are on in the emergency department during the day? _______
58. How many physicians are on in the emergency department during the night? _______
59. How many nurses/other staff are on in the emergency department during the day?
60. How many nurses/other staff are on in the emergency department during the night?
61. You have an adequate number of nurses in your emergency department.
   a. Strongly agree
   b. Agree
   c. Neutral
   d. Disagree
   e. Strongly disagree
62. You have an adequate number of physicians in your emergency department.
   a. Strongly agree
   b. Agree
   c. Neutral
   d. Disagree
   e. Strongly disagree
63. Consulting specialty physicians are available when needed.
   a. Strongly agree
   b. Agree
   c. Neutral
   d. Disagree
   e. Strongly disagree
64. The nurses in your emergency department are adequately trained.
   a. Strongly agree
   b. Agree
   c. Neutral
   d. Disagree
   e. Strongly disagree

65. The physicians in your emergency department are adequately trained.

a. Strongly agree
b. Agree
c. Neutral
d. Disagree
e. Strongly disagree

66. Training physicians to specifically staff an Emergency Department is beneficial to patient care.

a. Strongly Agree
b. Agree
c. Neutral
d. Disagree
e. Strongly Disagree

Medical Transportation Questions

67. Please estimate how patients get to the hospital:

a. Walk/carried ______%
b. Private car ______%
c. Taxi ______%
d. Police ______%

e. Public ambulance ______%

f. Private ambulance ______%

68. When a patient arrives, who brings the patient from the vehicle into the hospital's reception room?

69. It is important is it to have an ambulance service in my city.

a. Strongly agree
b. Agree
c. Neutral
d. Disagree
e. Strongly disagree

70. It is important to have trained medics and paramedics to transport patients.

   a. Strongly Agree
   b. Agree
   c. Neutral
   d. Disagree
   e. Strongly Disagree

71. What is the average time it takes for a patient in your area to get to the closest hospital in case of emergency?

   a. <5 minutes
   b. 5 – 30 minutes
   c. 31 – 60 minutes
   d. 61 – 120 minutes
   e. 121 – 180 minutes (2-3 hours)
   f. >3 hours

72. Is there a universal phone number to call to get an ambulance in your area?

   a. Yes
   b. No
   c. Not sure

73. If so, how reliable is this phone number?

   a. Very reliable
   b. Reliable
   c. Not sure
   d. Not reliable
   e. Almost never working

74. If you called this phone number, how long on average does it take an ambulance to arrive?

   a. <5 minutes
   b. 5 – 30 minutes
   c. 31 – 60 minutes
   d. 61 – 120 minutes
   e. 121 – 180 minutes (2-3 hours)
   f. >3 hours

75. If a family member became *seriously* ill at home, how would you seek medical care?
  a. Keep comfortable/treat at home
  b. Wait for a doctor to arrive at home
  c. Carry to the hospital
  d. Transport to the hospital via private car or taxi
  e. Call for an ambulance

76. If a family member became *seriously* ill outside the home, how would you seek medical care?
  a. Keep comfortable/take home
  b. Wait for a doctor to arrive at home
  c. Carry to the hospital
  d. Transport to the hospital via private car or taxi
  e. Call for an ambulance

Safety Questions

77. You feel safe at home.
  a. Strongly agree
  b. Agree
  c. Neutral
  d. Disagree
  e. Strongly disagree

78. You feel safe when commuting to work.
  a. Strongly agree
  b. Agree
  c. Neutral
  d. Disagree
  e. Strongly disagree

79. You feel safe when in the emergency department.
  a. Strongly agree
  b. Agree
  c. Neutral

    d. Disagree
    e. Strongly disagree

80. You feel safe when in other parts of the hospital (excluding the emergency department).
    a. Strongly agree
    b. Agree
    c. Neutral
    d. Disagree
    e. Strongly disagree

81. Have you ever been assaulted by a patient or patient’s family member when in the emergency department?
    a. Never (0)
    b. Once (1)
    c. Twice (2)
    d. 3 – 5 times
    e. 5 – 10 times
    f. 10 – 20 times
    g. >20 times

82. Have you ever had a patient or patient’s family member threaten you with a gun when in the emergency department?
    a. Never (0)
    b. Once (1)
    c. Twice (2)
    d. 3 – 5 times
    e. 5 – 10 times
    f. 10 – 20 times
    g. >20 times

83. Have any of your medical colleagues at your hospital been injured by violence at work in the last year?
    a. None (0)
    b. 1
    c. 2
    d. 3 – 5
    e. 5 – 10
    f. 10-20
    g. >20

84. Have any of your medical colleagues at your hospital been killed by violence at work in the last year?

a. None (0)
b. 1
c. 2
d.3 – 5
e.5 – 10
f. >10

85. The security in the emergency department is adequate.

a. Strongly agree
b. Agree
c. Neutral
d. Disagree
e. Strongly disagree

Equipment and Capacity Barriers to Emergency Care Questions

86. Necessary medications are immediately available for use during emergencies.

a.Strongly agree
b.Agree
c.Neutral
d. Disagree
e. Strongly disagree

87. Necessary equipment is immediately available for use during emergencies.

a.Strongly agree
b. Agree
c. Neutral
d. Disagree
e. Strongly disagree
