# Supplementary material for: A structured assessment of emergency and acute care providers in Afghanistan during the current conflict
Source: Int J Emerg Med. 2015 Jul 4;8:21. doi: 10.1186/s12245-015-0069-0 (PMC4495094; doi:10.1186/s12245-015-0069-0)
Supplement: Additional file 2: — Focus Group Questionnaire. [file 12245_2015_69_MOESM2_ESM.pdf]

Additional file 2
Focus Group Questionnaire

1- What are the major challenges to delivering healthcare in your profession?
2- What are the most pertinent or pressing issues within your hospital?
3- What are the major resource limitations/issues?
4- What are the major human resources issues you deal with?
5- What are the major training issues you deal with?
6- Do you feel that pre-hospital and EM care should be a priority in your hospital?
