# Supplementary material for: A structured assessment of emergency and acute care providers in Afghanistan during the current conflict
Source: Int J Emerg Med. 2015 Jul 4;8:21. doi: 10.1186/s12245-015-0069-0 (PMC4495094; doi:10.1186/s12245-015-0069-0)
Supplement: Additional file 3: — Summary Measures Defined. [file 12245_2015_69_MOESM3_ESM.pdf]

Additional file 3

Summary Measures Defined

	(Question #) /Question
Skill	(6)knowledge of BLS, ACLS, and ATLS
	(7)ever taken BLS training
	(8) ever taken ACLS training
	(9) ever taken ATLS training
	(10) have ER certification or diploma
	(14)ever performed endotracheal intubation
	(15) feel comfortable with intubation in an emergency
	(16) feel comfortable with the initial resuscitation of a medical patient
	(17) feel comfortable with the initial resuscitation of a trauma patient
Staff	(61)there are an adequate # of nurses in the emergency room
	(62)there are an adequate # of of MDs in the emergency room
Safety	(78)Feel safe getting to hospital
	(79)/feel safe in the emergency department
	(80)/feel safe in other parts of the hospital
	(85)/adequate security in the emergency department
Equipment	(86)-necessary medications are available in the emergency dept
	(87)- necessary equipment are available in the emergency dept
Assault	(81)-assaulted by a patient or patient's family member when in the emergency department?
	(82)-patient or patient's family member threaten you
	(83)-your medical colleagues at your hospital been injured by violence at work in the last year?
	(84)-your medical colleagues at your hospital been killed by violence at work in the last year?
Access	(71)-average time to the closest hospital
	(72)-Is there a universal phone number
	(73)-how reliable is this phone number
	(74)-how long on average does it take an ambulance to arrive
Prehospital Care/Transport time	(71)-average time to the closest hospital
	(74)-how long on average does it take an ambulance to arrive
Confidence in the health system	(19)-Patients will benefit from initial treatment in the emergency department
	(64)-nurses in your emergency department are adequately trained
	(65)-physicians in your emergency department are adequately trained
